# A frontier in the understanding of synaptic plasticity: Solving the structure of the postsynaptic density

**DOI:** 10.1002/bies.201200009

**Published:** 2012-04-23

**Authors:** Matthew G Gold

**Affiliations:** Department of Neuroscience, Physiology and Pharmacology, University College LondonLondon, UK

**Keywords:** mass spectrometry, memory, postsynaptic density, structure, synaptic plasticity

## Abstract

The postsynaptic density (PSD) is a massive multi-protein complex whose functions include positioning signalling molecules for induction of long-term potentiation (LTP) and depression (LTD) of synaptic strength. These processes are thought to underlie memory formation. To understand how the PSD coordinates bidirectional synaptic plasticity with different synaptic activation patterns, it is necessary to determine its three-dimensional structure. A structural model of the PSD is emerging from investigation of its molecular composition and connectivity, in addition to structural studies at different levels of resolution. Technical innovations including mass spectrometry of cross-linked proteins and super-resolution light microscopy can drive progress. Integrating different information relating to PSD structure is challenging since the structure is so large and complex. The reconstruction of a PSD subcomplex anchored by AKAP79 exemplifies on a small scale how integration can be achieved. With its entire molecular structure coming into focus, this is a unique opportunity to study the PSD.

## Introduction

How does the brain encode memories? Each new experience leads to a complex pattern of electrochemical communication between subpopulations of neurons that are interconnected by synapses. A popular theory is that changes in the strength of these synapses are an important component of memory formation 1: cellular protocols dictate the direction of synaptic plasticity depending on the stimulation pattern of each synapse. Influential supporting evidence for this theory is that different artificial stimulation protocols lead to either strengthening or weakening of synapses between CA3 and CA1 neurons in the hippocampus. High-frequency tetanic stimulation of Schaffer collateral axons projected by CA3 neurons leads to an increase in the excitatory postsynaptic potential elicited in CA1 neurons with which they form synapses. This effect lasts for several hours and is known as long-term potentiation (LTP), a term coined to describe a similar phenomenon in excitatory connections on hippocampal granule cells 2. Conversely, low-frequency stimulation leads to long-lasting weakening of the same synaptic population known as long-term depression (LTD) 3. Together, LTP and LTD at Schaffer collateral-CA1 synapses constitute the prototypical form of synaptic plasticity. The molecular basis of both LTP and LTD has been vigorously investigated on the assumption that it will uncover general mechanisms employed by neurons in modifying synaptic strength. This research has revealed that signalling in a proteinaceous specialisation of the dendritic spine called the postsynaptic density (PSD) is central to the induction of both LTP and LTD 4.

The PSD was first observed in electron micrographs as ‘localised regions of thickening and increased density’ 5 attached to the postsynaptic 6 membrane of excitatory synapses. The thickness of the PSD was first measured at ∼50 nm by electron microscopy (EM) of isolated PSDs 7. EM of thinly sectioned hippocampal neurons indicates that a PSD at the head of a typical ‘thin’ dendritic spine is disc shaped, with a surface area of ∼0.07 µm^2^ and a thickness of ∼25 nm 8, 9. The PSD positions glutamate receptors across from pre-synaptic glutamate release sites, and links the receptors to intracellular signalling cascades. The structure is a locus for mutations causing neurological disease and psychiatric disorders 10, underlining its critical role in synaptic transmission and plasticity. For example mutations in the PSD scaffold proteins Shank2 11 and Shank3 12 are associated with Autism Spectrum Disorders, while mutations in LGI1 and ADAM22 are related to epilepsy 13. Investigation of the molecular basis of frequency-dependent synaptic plasticity in the Schaffer collateral pathway has revealed some general mechanisms for synaptic plasticity: entry of Ca^2+^ into the PSD through NMDA-type glutamate receptors (NMDARs) is often a requirement for both LTP 14, 15 and LTD 3, 16, and trafficking and regulation of the open probability/single-channel conductance of AMPA-type glutamate receptors (AMPARs) is fundamental to changes in the strength of many types of synapse 17. However, the field awaits a mechanism to describe how signalling molecules in the PSD can link Ca^2+^ entry to both up or down-regulation of AMPAR currents depending on the pattern of stimulation. As I shall discuss, a number of signalling proteins are thought to modify AMPAR currents 18 in response to Ca^2+^ entry. However, many different signalling molecules have been implicated, and the PSD is so complex that it is difficult to be sure which molecules constitute the ‘core program’ 19 for induction of synaptic plasticity.

**Figure 1:**
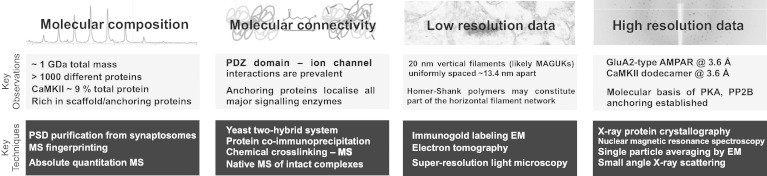
A multi-faceted experimental scheme for solving the structure of the PSD. Key observations and techniques are listed for four experimental branches that are enabling determination of the molecular structure of the PSD.

Linus Pauling opined that ‘It is structure that we look for when we try to understand anything’ 20. Structural studies have been integral to conceptual breakthroughs throughout the history of neuroscience. Examples include the development of neuron theory by Cajal on the basis of characterisation of the neuronal architecture of the brain using the Golgi method, and support for chemical transmission through resolution of the synaptic cleft by EM. Determination of the layout of ion channels and signalling molecules in the PSD at high resolution also has the potential to drive conceptual development. In this review, I shall discuss progress on four experimental branches that are necessary for a molecular reconstruction of the PSD (Fig. 1). The complexity of the structure is such that data integration and model building are crucial for progress, and these topics are also reviewed. Firstly though, I shall further consider the significance of PSD structure in research into the molecular basis of synaptic plasticity.

## The molecular structure of the PSD is a blueprint for understanding synaptic plasticity

Research into the molecular basis of synaptic plasticity has converged on the regulation of AMPAR phosphorylation by enzymes that respond to postsynaptic Ca^2+^ entry. During LTP, residue Ser831 of GluR1 AMPARs is phosphorylated by Ca^2+^/calmodulin (CaM)-dependent protein kinase II (CaMKII) and protein kinase C 21, 22, which increases single-channel conductance 23. Ser831 phosphorylation is opposed by phosphatases 21, which are thought to include protein phosphatase 1 acting downstream of Ca^2+^/CaM-activated protein phosphatase 2B (PP2B) 24. PP2B also directly dephosphorylates the cAMP-dependent protein kinase A (PKA) phosphorylation site Ser845 21 during LTD, which both decreases the open probability of AMPARs and leads to their trafficking out of the PSD. This AMPAR phosphorylation-centric model 25 of synaptic plasticity is supported by changes in hippocampal AMPAR phosphorylation in rats following inhibitory avoidance training 26.

**Figure 2:**
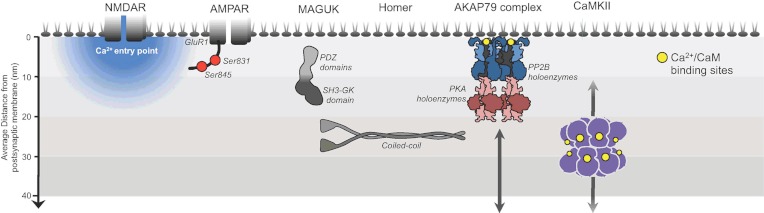
Laminar organisation of PSD signalling molecules involved in AMPAR phosphoregulation. Approximate distances are illustrated from the internal edge of the postsynaptic bilayer in the axodendritic axis. Distances are derived from immuno-EM 67, 70, 105 studies, with the exception of the AKAP79 complex, whose position is approximated on the basis that the N-terminus of AKAP79 binds to membrane phospholipids 106. Protein outlines of a MAGUK protein (SAP97), Homer, the AKAP79 complex and the CaMKII holoenzyme are approximately to scale 54, 68, 72, 84. The lateral organisation of proteins in the diagram is not intended to be realistic.

How then can high-frequency tetanic stimulation and low-frequency stimulation lead to opposite effects when both LTP and LTD are induced by Ca^2+^-sensitive enzymes acting downstream of postsynaptic Ca^2+^ entry? It was suggested that the direction of synaptic plasticity was dictated by the NMDA receptor subtype 27 present in a given PSD, but this has since been refuted 28. Rather than being determined by the presence or absence of a particular protein, it is likely that spatio-temporal subtleties in Ca^2+^ signals determine the direction of plasticity. Second messengers, including Ca^2+^ and cAMP, are elevated in cellular microdomains 29, 30; a second messenger-responsive enzyme will not be activated unless it is positioned within such a microdomain. Similarly, substrates must be in proximity to the microdomain or they will not be acted upon. Three factors that affect whether a second messenger-dependent signalling event falls within a microdomain are: (i) the size of the microdomain as determined by the signal amplitude, for example high-frequency tetanic stimulation enables maximal Ca^2+^ entry through NMDARs and thus activates signalling enzymes over a larger volume; (ii) the position of enzymes and substrates in relation to the second messenger generation/entry location, for example the anchoring protein AKAP79 (AKAP150/AKAP5) positions PKA and PP2B for bidirectional phosphoregulation of AMPAR GluR1 residue Ser845 31, 32; (iii) the duration of the elevation in second messenger concentration since the architecture of the signalling microdomain may itself be regulated by the second messenger 33. Importantly, the Ca^2+^/CaM-sensitive enzymes CaMKII and PP2B are probably positioned at different distances from the NMDAR mouth in the PSD axiodendritic axis (Fig. 2), which suggests that PP2B may be able to sense smaller quantities of postsynaptic Ca^2+^ entry. The significance of signalling enzyme targeting is underlined by many reports of protein-protein interactions critical to synaptic plasticity, including interactions that position CaMKII 34, 35. Therefore, to understand synaptic plasticity, it is necessary to determine how the critical Ca^2+^-sensitive signalling enzymes are positioned relative to Ca^2+^ entry points and their substrates in the PSD. Answering this question requires an accurate structural model of the PSD.

The PSD structure also provides a foundation for understanding how synaptic plasticity is expressed following induction. For example the machinery for endocytosis of AMPARs is positioned laterally to the PSD and connected to it by direct protein-protein interactions 36. Pre-synaptic changes also contribute to the expression of synaptic plasticity 37. The emphasis in this review is on understanding the canonical homosynaptic frequency-dependent form of synaptic plasticity in the Schaffer collateral pathway. However, PSD structure is also likely to contribute to the understanding of forms of plasticity that are pre-synaptically induced, such as in the mossy fibre pathway 38, 39, by explaining how signalling processes operate through PSD proteins that cross the synaptic cleft; and that are induced by different stimulation protocols, such as spike-timing dependent plasticity 40. Given that it is critical to form a structural model of the PSD, the following sections describe the progress and outlook of four experimental branches that enable the molecular structure of the PSD to be pieced together (summarised in Fig. 1).

## Determination of the molecular composition of the PSD is fundamental to its reconstruction

The starting point for assembling a structural model of the PSD is the identification and quantification of its constituent molecules. Methods to purify the PSD are an essential component of such research. PSD purification protocols are predominantly derived from the Whittaker synaptosome preparation 41, in which a sucrose gradient is applied to a particulate brain fraction to purify the nerve endings. PSDs can be isolated from synaptosomes by extracting the synaptic membranes with non-ionic detergent 42 such as 1% Triton X-100 43.

The first PSD proteins were identified by biochemical characterisation of the most prominent proteins 44 following SDS-PAGE of purified PSDs, such as CaMKII 45 and the membrane-associated guanylate kinase (MAGUK) protein PSD-95 46. Latterly, peptide mass fingerprinting by mass spectrometry (MS) has revealed that a great diversity of different proteins are located in the PSD. For example 1,461 proteins were identified by this approach in PSDs purified from human neocortex 10. Comparison of different proteomic efforts shows that more than 400 proteins are regularly identified across different PSD preparations 47 by MS fingerprinting. Membrane receptors and channels, proteins involved in signalling by protein phosphorylation and scaffold and anchoring proteins are well represented in the PSD proteome 48. Within the scaffold protein subset, proteins containing PDZ domains are a hallmark of the PSD.

The presence of a large subset of mitochondrial proteins in the ‘PSD’ proteome 48 is cautionary. While identification of PSD proteins by MS fingerprinting is extremely sensitive, the efficacy of the approach is limited by the quality of the initial PSD purification. Limitations of the current PSD purification method include: cellular contaminants including mitochondria, loss of PSD proteins in high concentrations of detergent during the membrane extraction phase and presence of pre-synaptic proteins via physical association with PSD proteins that bridge the synaptic cleft. PSD purification methods are essentially unchanged in over 30 years 10, 42, so there may be scope for improvement in this area.

Although the number of proteins identified in the PSD is daunting, the dimensions of the PSD limit the diversity of proteins that can be accommodated within it. The Matthew's coefficient V_M_, which is used to assess the solvent content of protein crystals 49, can be used to estimate the mass of the PSD from its volume. The most commonly observed values for V_M_ in protein crystals cluster around 2.15 Å^3^/Da, which corresponds to 43% solvent content. If we assume that the PSD is closely packed to the same degree, for a typical PSD with a volume of ∼2 GÅ^3^ the estimated protein mass is 0.93 GDa. This is consistent with a mass of ∼1 GDa measured by scanning EM of purified PSDs 50, suggesting that ∼15–20,000 polypeptide chains make up a single PSD.

Some proteins are present at a high-copy number in the PSD, which simplifies the problem. The MS ‘absolute quantification’ method, which uses labelled synthetic peptides as internal standards, has been utilised to determine the concentrations of a selection of proteins in PSDs purified from rat forebrain 51. CaMKII isoforms were found to constitute ∼9% of total PSD protein by this method 51. These concentrations can be used to estimate the copy numbers of different proteins in the PSD – approximately 5,600 copies in the case of CaMKII 48, 51. Scaffold proteins are also over-represented, with ∼300*PSD-95, 360*SynGAP, 150*Shank isoforms and 20*AKAP79 per PSD 48. Copy numbers of PSD proteins determined by both quantitative gel electrophoresis 50 and a GFP-based calibration technique 52, are generally consistent with these numbers. Comparative analysis using cleavable isotope-coded affinity tag MS shows that there are regional differences in the protein make-up of the PSD. For example SynGAP and CaMKII are expressed approximately five-fold more highly in PSDs purified from forebrain compared to cerebellum 51. Isobaric tagging MS has enabled comparative analysis of protein expression and phosphorylation in the murine cortex, midbrain, cerebellum and hippocampus 53. This revealed that protein phosphorylation is relatively higher in the hippocampus. Regional differences in PSD composition reflect regional variations in the molecular mechanisms underlying synaptic plasticity 18. Nevertheless, the majority of proteins compared in forebrain and cerebellum were not expressed at significantly different levels 51. This suggests that PSDs perform broadly similar functions throughout the brain.

Developing methods are useful for determining the stoichiometries of protein subcomplexes present in the PSD. Mass calculation of intact protein complexes by native MS can be sufficiently accurate to enable unambiguous stoichiometric assignment. A PSD subcomplex nucleated by AKAP79 was measured at 466 kDa by this method, indicating that the complex consists of two copies of AKAP79, two copies of CaM, four copies of PKA and four copies of PP2B 54. The single-molecule pull-down assay 55, which relies on fluorescence microscopy and can be applied in vivo, is also likely to be useful in determining the exact stoichiometries of PSD subcomplexes. Continued characterisation of the PSD proteome by quantitative techniques will facilitate model building by establishing how many copies of each PSD protein must be positioned within global structural models.

## The molecular connectivity of the PSD limits how its molecular components can be assembled

Order in the PSD arises from specific interactions between surfaces presented by its constituent molecules. Determining these interactions is an important step in reconstructing the PSD. Protein-protein interactions were initially identified in the PSD by yeast two-hybrid screening. Examples are the interaction between the C-terminus of NR2B-type glutamate receptors and the second PDZ domain of PSD-95 56, and the AKAP79-PP2B interaction 57. SynGAP was identified by this approach using the third PDZ domain of a MAGUK protein as bait 58.

The yeast two-hybrid system is less well suited to identifying lower affinity interactions found in multi-protein complexes in which each protein simultaneously interacts with multiple proteins. These interactions are a feature of the PSD. An alternative approach is to immunoprecipitate a given PSD protein and characterise its co-precipitants. For example a MAGUK-associated signalling complex containing approximately 100 proteins can be purified using either the last six C-terminal amino acids of NR2B as an affinity ligand, or with anti-NR2B antibodies 59, 60. The co-immunoprecipitating proteins are likely to be in proximity to each other. Immunoprecipitations are often performed using whole brain lysate. In the case of NMDAR-associated proteins, care must be taken to distinguish proteins in complex with synaptic and extra synaptic NMDARs, which have important functional differences 61. Variations on the co-immunopreciptation strategy include the use of low-background tandem affinity purifications, such as was used to determine PSD proteins in complex with PSD-95 62.

Co-immunoprecipitation does not distinguish direct and indirect interactions. Follow-up experiments with purified protein and methods to quantify binding thermodynamics such as isothermal titration calorimetry can differentiate between the two interaction modes. However, weaker direct PSD interactions may still evade detection outside of the context of the PSD. A novel approach is peptide fingerprinting of cross-linked peptides following trypsinisation of cross-linked protein complexes 63. In this method, protein complexes are chemically cross-linked with a cross-linker of a set linker length prior to trypsinisation. If the spectrometer detects a molecule corresponding to peptides from two different proteins bridged by the cross-linker this indicates that the cross-linked residues from the two proteins are within a certain distance in the native structure 64, 65. The approach has been applied on a small scale to identify a homomeric dimerisation site in AKAP79 in proximity to residues 328–333 54. It could be applied on a larger scale to map interactions in PSD subcomplexes or within an intact PSD. Analysis of fragmentation patterns in native MS experiments is another avenue for determining the connectivity of proteins within multi-protein complexes 66. These novel MS-centric approaches are likely to complement the current methods for determining PSD connectivity in the future.

## Electron and super-resolution light microscopy are revealing PSD structure at low resolution

EM continues to play an important role in interrogating global PSD structure, following the first imaging of the PSD by this method in the 1950s. Structures as small as 3–4 nm in diameter can be resolved within 1–1.5 nm-thick virtual sections following electron tomography of dendritic spines 67. This approach reveals that ‘vertical’ filaments approximately 5 nm wide and 20 nm long are uniformly spaced throughout the PSD with a nearest neighbour distance of 13.4 nm. At this spacing roughly 400 vertical filaments punctuate a PSD 400 nm in diameter. Comparison with single-particle images of the MAGUK protein SAP97 by EM indicates that the vertical filaments are approximately the length of an extended MAGUK protein 68. Immunogold labelling studies, and the patchy loss of vertical filaments 69 in PSD-95 knockdown neurons, suggests that MAGUK proteins with their N-termini proximal to the membrane correspond to vertical filaments. Laminar organisation is a general feature of the PSD, with the peak concentrations of different proteins at different distances from the membrane 70 (Fig. 2). Classification of AMPA and NMDA-type glutamate receptors as well as horizontal and vertical filaments has enabled construction of a model of the core filamentous structure of the PSD 67. PSD horizontal filaments fall into two morphological classes, which may correspond to the scaffold proteins GKAP and Shank 67. Negative stain EM demonstrates that Shank3 is filamentous 71 and that a 1:1 mixture of purified Homer1b and Shank1C polymerises to form a network structure. Therefore, Homer and Shank may constitute an important component of the horizontal filament network 72. Immunogold labelling also reveals that the endocytic machinery (AP-2, clathrin and dynamin) is sequentially organised laterally to the PSD in dendritic spines 73. This suggests that receptors decouple from the PSD prior to endocytosis in an adjacent functional domain. Additional ultrastructural features, including filaments linking the PSD and actin cytoskeleton 74, are visible following high-pressure freezing. This approach likely produces more realistic images than normal aldehyde fixation.

‘Super-resolution’ light microscopy techniques, based on single-molecule detection, are contributing to investigations of PSD ultrastructure 75, 76. Spatial resolution approaching 20 nm has been achieved using photo-activated localisation microscopy (PALM) 76 and the related stochastic optical reconstruction microscopy (STORM) 77. The axial distributions of synaptic proteins measured using STORM 77 are broadly consistent with prior immuno-EM studies 70. Two advantages of PALM/STORM compared to EM are the ability to observe dynamics and to simultaneously visualise multiple molecules. These were showcased in a recent study of actin spine dynamics 78. Quantum dot imaging is another powerful method for following in vivo dynamics at high resolution, for example this approach has been applied to show that GluR1 AMPAR mobility is restricted in active synapses 79. Super-resolution light microscopy, in tandem with two and three-dimensional EM approaches, should enable a three-dimensional map of constituent proteins to be assembled at lower resolution.

## Analysis of purified protein substructures reveals PSD structural features at high resolution

A low-resolution model of the PSD could be drawn up using information gathered by the techniques outlined in the three preceding sections. In order to add molecular detail, high (∼3 Å or better) resolution structural information is required. Progress has been made in determining high-resolution structures of the proteins that comprise the AMPAR phosphorylation-centric model of bidirectional synaptic plasticity 49. The crystal structure of the transmembrane and extracellular domains of the GluA2 AMPAR has been determined 80. Crystal structures of the ligand-binding domains of the NMDAR 81 provide insights into the arrangement of the receptor subunits. The structure of the NMDAR pore, which corresponds to the entry sites of Ca^2+^ into the PSD, is yet to be determined. The three-dimensional structures of the globular signalling enzymes that control the phosphorylation state of AMPA receptors have been solved 82–85. These include a crystal structure of the majority of the PKA holoenzyme in which the cAMP-binding domains of the RII subunit form multiple contact points with the catalytic subunit 82; and the CaMKII holoenzyme structure showing that the CaM-binding sites are inaccessible in the auto inhibited kinase dodecamer 84.

High-resolution structures detailing PSD protein-protein interactions are particularly constructive. MAGUK family proteins, which simultaneously interact with multiple integral membrane proteins via PDZ domain interactions, are essential for the structural integrity of the PSD 69. The molecular basis of selective PDZ domain interactions has been established 86. Further important interactions that have been determined at high resolution include the anchoring mechanisms for PKA and PP2B. AKAP79 associates with the N-terminal dimerisation and docking (D/D) domain of PKA RII subunits through a hydrophobic interface presented by an amphipathic helix that is conserved in the AKAP family 87, 88. An additional structural motif in AKAP79 simultaneously anchors two copies of PP2B 54. The anchoring protein contributes the central strand of a three-stranded β-sheet, in which the outer β-strands correspond to β-14 strands of PP2B catalytic A subunits 89, 90. It can be difficult to obtain high-resolution structures of full-length anchoring and scaffold proteins, which are generally too large for nuclear magnetic resonance and lack tertiary structure elements that favour crystallisation. Crystallising protein complexes is also challenging. In such cases complementary techniques including small angle X-ray scattering 84 and single-particle averaging by EM can generate electron density maps at a resolution that is at least sufficient to locate protein subdomains 68. These approaches may also reveal large-scale dynamics, for example between the PDZ and SH3-GK domains of SAP97 68.

For PSD proteins where no high-resolution structure is available, structure can potentially be modelled by homology to proteins of known structure. If not, application of the battery of approaches detailed above is likely to yield useful information.

## Integration of PSD structural data into inclusive models will present functional insights

**Figure 3:**
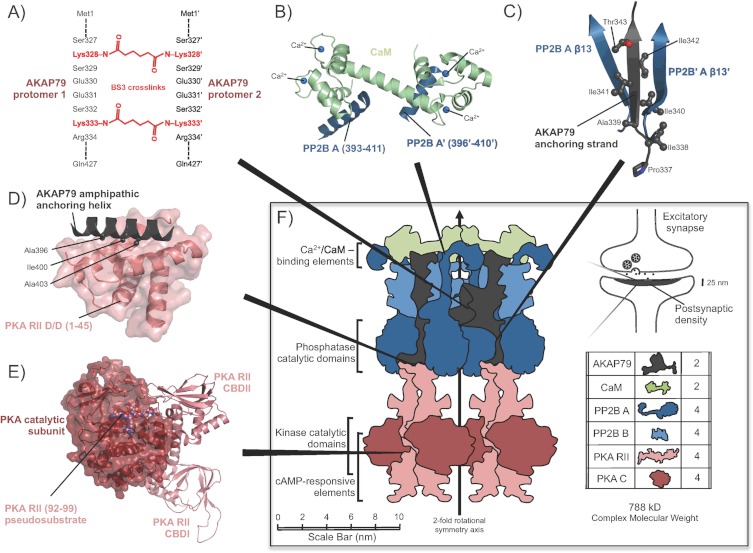
Structure of the postsynaptic AKAP79 signalling complex. A: AKAP79 homomeric cross-linking sites indicate that AKAP79 forms a parallel dimer and that there is likely to be a dimerisation site in proximity to residues 328–333 54. B: Crystal structure of a complex between PP2B (blue) and CaM (green; PDB ID 2R28) 107. C: Crystal structure of the AKAP79 phosphatase anchoring peptide (grey) in complex with two PP2B A subunits (blue; PDB ID 3LL8) 90. D: Molecular basis of PKA anchoring to AKAP79. AKAP79 presents an amphipathic helix (grey) for interaction with a hydrophobic face on the PKA RII D/D domain (pink; PDB ID 2IZX) 87. E: Crystal structure of a complex between PKA RII (light red) and C (dark red) subunits (PDB ID 2QVS) 82. F: The stoichiometry of an intact 2*AKAP79:2*CaM:4*PP2B:4*PKA complex was determined by native mass spectrometry 54. In the central model, protein outlines and the scale bar are derived from crystal structures with the exception of AKAP79, the C-terminal 150 amino acids of PP2B A and the linker (residues 46–90) between the PKA RII D/D and cAMP-binding domains.

Francis Crick thought that ‘A good model… should serve to unite evidence from several different approaches’ 91. In the case of the PSD, information concerning its structure continues to accumulate from different approaches. Perhaps the greater challenge is uniting the data into a comprehensive model. The postsynaptic AKAP79 signalling complex 31, 32 exemplifies on a relatively small scale how different types of structural information can be integrated. In this case we know that its interacting proteins include PP2B, PKA and CaM; the location of its dimerisation site (Fig. 3A); the structures of associated proteins PKA, PP2B and CaM at high resolution; and the structures of interfaces between PP2B-CaM (Fig. 3B), AKAP79-PP2B 89 (Fig. 3C), AKAP79-PKA 87, 88 (Fig. 3D) and PKA RII and C subunits (Fig. 3E) at high resolution. Given the stoichiometry of the complex, which was measured using native MS 54, we can draw up the model shown in Fig. 3F. The model suggests that the complex is approximately 20 nm in its longest dimension, and likely spans the depth of the PSD (Fig. 2). It also suggests that the Ca^2+^/CaM-sensitive elements of AKAP79 and PP2B are proximal to the membrane, whereas the cAMP-binding elements of PKA are distal to the membrane, with the catalytic sites of anchored enzymes PKA and PP2B in-between.

Evidence from different approaches would enable the model to be improved: If an electron density map of the complex could be determined this would allow docking of the respective crystal structures, as has been achieved with other multi-protein complexes 92. Absolute quantitation MS estimated that approximately 20 copies of AKAP79 are present per PSD 51; determination of the location of these complexes within the PSD by a technique such as PALM would enable extension of the model to the scale of the PSD. Finally AKAP79 is known to interact with MAGUK proteins 93, but this interaction is poorly characterised. With the molecular basis of this interaction in hand, the model could be integrated with structural models of MAGUK proteins in the PSD. Given that the AKAP79 signalling complex constitutes 788 kD, the presence of ∼20 copies of AKAP79 per PSD, and an estimated mass of ∼1 GDa per PSD, the AKAP79 signalling complex may contribute as much as ∼1.6% of the total mass of the PSD. Reconstructing the PSD from discrete PSD subcomplexes such as the AKAP79 complex is one way to make PSD complexity more manageable.

Bioinformatics tools and resources can facilitate PSD model building (Table 1). The Genes to Cognition server details the content of different PSD proteomes and PSD subcomplexes 10, and public databases of mammalian protein-protein interactions include the STRING database 94. The worldwide databank (wwPDB) is valuable for sharing larger models of PSD structure that incorporate data collected using EM 95. Freely available programs for docking crystal structures into electron density maps 96–98 can assist construction of these models. There is a general trend in molecular biology from reductionism towards synthesis 99 reflected in the burgeoning field of systems biology. Signalling in the PSD is an appropriate subject for systems biologists, since the field is at the point where many different signalling molecules have been implicated but how they function together is poorly understood.

**Table 1:** Bioinformatics for integrating data to develop structural PSD models

Category	Tool/resource	Capabilities
Databases	G2Cdb	The Genes to Cognition consortium's database includes proteins identified by mass spectrometry in complex with the mouse NMDA receptor, present in the mouse postsynaptic proteome and present in PSDs isolated from human neocortex 10
	STRING	Extensive database of known and predicted protein interactions 94
	wwPDB	The Worldwide Protein Data Bank consists of the major international organisations for archiving macromolecular structural data, including the RCSB PDB. The EM Data Bank is to join the archive in 2012 95
Utilising atomic coordinates and electron density maps	Chimera	Extensible program for visualisation, analysis and editing of molecular structures, well suited to handling supramolecular assemblies and manually positioning crystal structures in electron density maps 96
	CCP4 suite	Includes programs for atomic model building (Coot) and exploration of macromolecular interfaces, surfaces and assemblies (PDBePISA) 97
	NORMA	Automated flexible fitting of high-resolution protein structures into electron-microscopy-derived electron density maps 98
Physiological modeling	CellDesigner	Models of gene-regulatory and biochemical networks, based upon differential equations, are stored using the versatile Systems Biology Markup Language (SBML). Provides support for graphical model construction 100
	NEURON	Enables kinetic modelling of ion concentrations, membrane voltage, and ion channels in compartmentalised neurons 109

Examples of freely available tools and resources are listed in three categories. Tools for physiological modelling and deep curation of protein-protein interactions were recently subject to an exhaustive review 108.

Computational modelling is an integral part of systems biology and the field is being driven by the development of new programs for modelling cellular systems 100. However, the most advanced network models will fail to reveal the underlying biology unless they closely resemble the structures that determine the attributes of their functional networks 101. Therefore, PSD structure can serve as a template for development and analysis of dynamic models of PSD signalling. Dynamics are an important consideration when constructing PSD structural models. Many proteins translocate into and out of the PSD, including AKAP79 102, and dynamics within the PSD must also be considered 103. It is likely that PSD horizontal and vertical filaments provide a framework for signalling enzymes of relatively greater mobility. The rearrangement of signalling microdomains over time following Ca^2+^ entry through NMDARs may reposition the key signalling enzymes CaMKII and PP2B in relation to critical substrates such as AMPARs. The best dynamic models of PSD signalling will also account for the effects of changes in membrane potential 101, such as depolarisation-induced release of NMDAR Mg^2+^block 104. Better structural models of the PSD should also provide insight into how mutations of PSD proteins pathologically affect synaptic signalling 11, 12.

It should be acknowledged that there are limitations to research targeted at PSD structure. PSDs are not truly unitary, as demonstrated by the heterogeneity in the composition of cerebellar and forebrain PSD preparations 51. Since the Schaffer collateral pathway is the canonical pathway for synaptic plasticity, it is logical to focus studies of PSD structure on the PSD located in the dendritic spines of CA1 neurons. It would be particularly helpful to have quantitative proteomic information relating to this PSD type. As discussed, PSD structure is dynamic 33, 78, 79, 102, 103. One solution to this complication is to attempt to ‘capture’ the PSD in different structural states, for example before and after prolonged Ca^2+^ elevation.

Finally, one must consider what level of accuracy is necessary for structural models to become functionally useful. Francis Crick provided this scientific parable in criticising a rival's model: ‘Why then was his model of so little use? …The reason is that his model did not approximate the real thing closely enough’ 91. Schematic and topographic models that are commonly presented in relation to PSD function tend to bear little similarity to realistic dimensions and often deal with only a selection of signalling molecules. Such models are likely to fall into the Crickian category of ‘existence proofs’ that do not lead to the generation of testable theories 91. We should continue to improve models that attempt to incorporate all PSD molecules in three dimensions until they are ‘close enough’ to reality to generate hypotheses that lead to deeper functional understanding. Given the progress of research in the four areas described in this review, this moment could soon be upon us.

## Conclusions

Evidence from four experimental branches is bringing the molecular structure of the PSD into focus. MS fingerprinting and quantitative analysis of PSD proteomes has revealed that, although a great diversity of proteins are accommodated within the PSD, some proteins are present at very high-copy numbers. Novel MS approaches are enabling a shift to a quantitative description of PSD composition. More systemic analysis of protein-protein interactions by MS analysis of cross-linked protein complexes is set to complement current approaches to characterising PSD protein-protein interactions. Structural proteins have been tentatively assigned to filaments running vertically and horizontally through the PSD and PALM/STORM will accelerate characterisation of the PSD ultrastructure at lower resolution. High-resolution crystal structures have been determined for several proteins with key roles in synaptic plasticity such as the NMDAR and CaMKII. High-resolution structures of PSD subcomplexes are very constructive, and progress is being made in this direction assisted by docking of crystal structures in electron density maps generated by single-particle averaging cryo-EM and small-angle X-ray scattering. Development of programs that enable dynamic systems biology modelling of complex signalling networks will aid integration of PSD structural data and the development of new theories to explain the molecular basis of synaptic plasticity.

As our understanding of the molecular structure of the PSD improves, it is likely to reveal mechanisms that provide deeper explanations of molecular processes at the synapse. Most significantly the structure provides a framework for understanding signalling in the induction of bidirectional synaptic plasticity, which is thought to enable information storage in the brain. Piecing together PSD structure is a challenge that should appeal to structural biologists who are redirecting their research towards the study of multi-protein complexes 101; and to systems biologists looking to model complex signalling networks of profound functional importance. The field is at a transition point, with different types of structural data approaching synthesis: this is an exciting time to be involved in structural biology of the PSD.

## Abbreviations

AMPAR: AMPA-type glutamate receptor
CaM: calmodulin
CaMKII: Ca^2+^/CaM-dependent protein kinase II
EM: electron microscopy
LTD: long-term depression
LTP: long-term potentiation
MAGUK: membrane-associated guanylate kinase
MS: mass spectrometry
NMDAR: NMDA-type glutamate receptor
PALM: photo-activated localisation microscopy
PKA: protein kinase A
PP2B: protein phosphatase 2B
PSD: postsynaptic density
STORM: stochastic optical reconstruction microscopy
